# Combinatorial protection of cochlear hair cells: not too little but not too much

**DOI:** 10.3389/fncel.2024.1458720

**Published:** 2024-09-17

**Authors:** Arwa Kurabi, Kwang Pak, Eun Jung Lee, Allen F. Ryan

**Affiliations:** ^1^Department of Otolaryngology, University of California San Diego, La Jolla, CA, United States; ^2^Department of Otorhinolaryngology-Head & Neck Surgery, Jeonbuk National University School of Medicine, Jeonju, Republic of Korea; ^3^Department of Neurosciences, University of California San Diego, La Jolla, CA, United States; ^4^San Diego Veterans Administration Healthcare System, La Jolla, CA, United States

**Keywords:** cochlea, ototoxicity, hair cell protection, multi-target treatment combination, *in vitro* model culture system, otoprotection

## Abstract

**Background:**

A number of drugs are toxic to the cochlear sensory cells known as hair cells (HCs), resulting in hearing loss. Treatment with survival-promoting growth factors, antioxidants, and inhibitors of cell death pathways or proteinases have been shown to reduce HC damage in *in vivo* and/or *in vitro* animal models. Conversely, translation to humans has often been disappointing. This may be due to the complexity of intracellular damage processes. We hypothesized that combining treatments targeting different cellular processes would be more effective.

**Methods:**

Using an *in vitro* model of gentamicin ototoxicity for murine cochlear hair cells, we screened all 56 possible combinations of inhibitors targeting five different cell damage mechanisms, plus the activator of one cell survival pathway, each of which have been shown to be singly effective in preventing HC loss in experimental studies. A high dose of gentamicin (200 μM) was used over three days in culture. All compounds were added at a dosage below that required for significant protection in the assay, and only this single dose was then employed. This was done so that we could more easily detect interactive, as opposed to additive, effects.

**Results:**

Increasing protection of hair cells was observed as combinations of compounds were increased from two to four factors, although not all combinations were equally protective. The optimal combination of four compounds consisted of an anti-oxidant, an apoptosis inhibitor, an autophagy inhibitor and a protective growth factor. Increasing the number of factors to five or six resulted in decreased protection.

**Conclusion:**

The results support the hypothesis that targeting multiple cellular damage or survival pathways provides more an effective hair cell protection approach. The results help to identify critical interactions among the cellular processes that operate in gentamicin ototoxicity. They also suggest that inhibiting too many biological processes impairs functions critical to HC survival, resulting in decreased protection.

## Highlights

- The process of damage to cochlear sensory cells is complex, yet most prevention studies target only a single damage process.
- We studied all 56 combinations of compounds targeting five distinct cellular mechanisms for protection against an ototoxin applied *in vitro*.
- Increasing the number of compounds increased the level of sensory cell protection up to four, but protection declined with five or six.
- The results implicate reactive oxygen species, apoptosis and cell proliferation as key damage mechanisms, while cell survival factors are also important.

## 1 Introduction

The loss of hearing is a common disorder that can be caused by exposure to loud sounds or certain drugs, aging, infections or genetics (Cunningham and Tucci, 2017). Severe hearing loss can cause substantial decreases in the quality of life (Punch et al., 2019), social interaction (Bennett et al., 2022) and employability (Graydon et al., 2019). The prevention of hearing loss is thus an important concern. Changes in hearing thresholds are typically due to loss of cochlear sensory cells, known as hair cells (HCs) due to their ciliary arrays. HCs translate the mechanical vibrations of sound into neural impulses that activate the central auditory system. Protecting these cells is an important key to the preservation of hearing.

A number of cellular processes that participate in HC damage have been identified by studies in animals or cell lines. Evidence exists for the involvement of reactive oxygen species (ROS) (Seidman et al., 2004; Kamogashira et al., 2015), stress kinase signaling (Ryals et al., 2017), Ca^++^ regulation (Esterberg et al., 2014), inflammation (Frye et al., 2019) and apoptosis (Wu et al., 2020). Experimental inhibition of each of these processes has been shown to protect HCs *in vitro* and/or *in vivo*. For adult mammalian HCs, which are post-mitotic, the induction of cell division can also induce cell death (Liu and Zuo, 2008). Survival-promoting growth factors can protect HCs from damage (Yamahara et al., 2015). The laboratory successes of HC protection have led to clinical trials to reduce hearing loss. However, translation to humans has had mixed results (Sha et al., 2006; Kocyigit et al., 2015; Suckfuell et al., 2014; Bagger-Sjoback et al., 2015; Bai et al., 2022; Orgel et al., 2023). Even successful trials in humans have generally been less effective than in animal experimental models.

There are multiple reasons for this discrepancy, which is all too frequent in the translation of laboratory results to clinical practice (Fernandez-Moure, 2016). Animal and *in vitro* models of HC protection involve carefully controlled damage and drug delivery, while patients often have variability in both damage and delivery of drugs to the cochlea (Nyberg et al., 2019). HC damage in humans often occurs over longer time periods than in experimental studies. Species differences in drug responses between humans and animals may play a role. In addition, most treatments employed in clinical trials address a single cellular process or pathway. However, damage to cells generally involves multiple parallel, overlapping and competing intracellular processes (Weng et al., 1999; Bhalla, 2003). The various cellular interactions can act in a manner similar to a microprocessor, integrating pathway outputs to determine cell fate. Inhibiting one damage process, or boosting a single survival pathway, may therefore be insufficient to protect HCs from damage. While many of the determinants of translation failure are difficult to address, the complexity of cellular damage can be approached by combining treatments targeting different involved processes (Chen and Lahav, 2016).

We previously performed several *in vitro* screens of different cellular damage processes using primary mammalian hair cells. These screens employed compound libraries of antioxidants, protein kinase inhibitors, autophagy inhibitors, proteinase inhibitors and phosphatase inhibitors for protection against gentamicin ototoxicity (Ryals et al., 2017; Noack et al., 2017; Hur et al., 2018; Lim et al., 2018; Draf et al., 2021). In each case, only a small fraction of antioxidants or inhibitors proved to provide protection, and most of those were only partially effective. Many other laboratories have also identified treatments that provide experimental HC protection, as reviewed above.

We reasoned that combining treatments identified by our and other groups’ experiments could enhance HC protection. We identified five damage processes for combinatorial inhibition: ROS, stress protein kinases, autophagy, apoptosis and cell division. In addition, we also integrated protection by survival-promoting growth factors. We identified six compounds with demonstrated HC protective activity for each of these processes, and tested the various combinations in an *in vitro* murine HC assay of gentamicin ototoxicity to determine if they provide better protection to hair cells compared to each compound used alone. All possible combinations of the six compounds are tested to evaluate the impact of their addition on the hair cell survival from gentamicin-induced damage, demonstrating potential interactive effects that could inform therapeutic strategies.

## 2 Materials and methods

### 2.1 Ethics statement

Experiments were performed to National Institutes of Health (NIH) guidelines for the safe and humane treatment of animals. The study was approved by the Institutional Animal Care and Use Committee of the San Diego VA Medical Center.

### 2.2 Animals

All studies were performed on transgenic mice in which the expression of eGFP was driven by an 8.5 kb *pou4f3* promoter construct (Masuda et al., 2011). This strain expresses eGFP, that in the organ of Corti, the cochlear sensory epithelium, is restricted to both inner and outer HCs.

### 2.3 Explant preparation

The organ of Corti was micro-dissected from the cochleas of 3–5 day old *pou4f3*/*eGFP* mouse pups. The apical portion of the epithelium, which is much less sensitive to ototoxicity, was discarded. The basal and middle turns, which we have found respond similarly to the high dosage of gentamicin employed, were divided with a diamond scalpel into micro-explants, each consisting of ~20 inner HCs and the associated outer HCs, totaling ~80 HCs in each assay. Explants were plated singly into the wells of flat-bottom 96-well plates, in media consisting of DMEM:F-12 plus Pen/Strep and 5% FBS. Streptomycin was kept below the HC damage threshold. DMSO was included at 0.1%, necessary to enhance the cell permeability of some compounds. Explants were allowed to attach to the wells for 24 h.

### 2.4 Compound combinations

The antioxidant (AO) chosen for the study was N-acetyl choline (NAC), which has been reported to offer protection in animal and human studies (Bai et al., 2022; Orgel et al., 2023; Feghali et al., 2001). The kinase inhibitor (KI) was *C. difficile* toxin B (CDTB), which inhibits upstream kinase elements of the Jun kinase (JNK) stress pathway (Bodmer et al., 2002). The cell proliferation inhibitor (PI) was fascaplysin, which blocks the protein expression of CDK4 and cyclin D1 (Soni et al., 2000). The calcium channel blocker (CI) was nimodipine, which stabilizes L-type calcium channels in their inactive conformation (Bean, 1984). The apoptosis inhibitor (AI) was ZVAD-FMK, which irreversibly binds the catalytic site of caspases (Van Noorden, 2001). The growth factor (GF) used was insulin-like growth factor-1 (IGF-1), known to promote HC survival (Yamahara et al., 2015). The concentrations employed for each compound were just below the threshold for HC protection when used alone (see Table 1).

**Table 1:** The six compounds evaluated and their combination assays screening concentration.

Class	Acronym	Name	Concentration
Antioxidant	AO	N-acetyl choline (NAC)	5.0 mM
Kinase inhibitor	KI	CDTB	1.0 ng/mL
Calcium channel blocker	CI	Nimodipine	10 nM
Proliferation inhibitor	PI	Fascaplysin	500 nM
Apoptosis inhibitor	AI	ZVAD-FMK	5.0 μM
Growth Factor	GF	IGF-1	10 nM

Concentrations were just below the threshold for HC protection when used alone. The compounds target five specific damage processes and include survival-promoting growth factor.

### 2.5 Combination testing

Negative and positive control explants were placed into culture in media alone for 24 h to allow for attachment (Day 0). Negative control explants then remained in media for an additional 72 h (Days 1–3). Positive control explants were then treated for three days with 200 μM gentamicin. Experimental explants treated with compound combinations were pre-treated with the combination during the 24 h attachment period (beginning on Day 0). They were then treated with the combination plus gentamicin at the beginning of Day 1, and cultured for three days (Days 1–3). Media for all conditions included 0.1% DMSO. Culture media were not changed after Day 0.

Cultures were conducted in triplicate, as for our prior compound screens (Ryals et al., 2017; Noack et al., 2017; Draf et al., 2021). Different ototoxin batches produced different levels of HC damage in our positive controls and presumably for our compound combinations, while some negative control explants varied in their survival over days. This required verification that control conditions were consistent plate-to-plate. Thus, negative and positive controls were included in each 96-well plate and were used for analysis of that plate’s compound treatment results.

GFP-positive cells were imaged by fluorescence microcopy at the end of the attachment period on Day 0, and at the end of Days 1, 2 and 3 post gentamicin treatment. Any micro-explant that did not attach and flatten in the well after attachment was excluded and repeated, since HC counts could not be accurately quantified at that time. HC counts, including both inner and outer HCs, were evaluated in ImageJ by two independent observers blinded to treatment. Counts on Days 1–3 were compared to the number of HCs present at the end of Day 0, prior to gentamicin treatment, by ANOVA and *t*-test with Bonferroni correction. The counts were then converted to percent of HCs present at the end of Day 0. HC survival curves were generated for both controls and for each experimental compound combination.

### 2.6 Data analysis

Comparisons of treatment counts to matching negative or positive control counts on the same culture plate were performed by *t*-tests with Bonferroni correction for multiple tests. Significant differences were replicated for confirmation. Statistical comparison across the different combinations were not possible, due to variability in control values between plates. Comparisons mentioned below are observational only. HC counts were converted to percentages, compared to those present at the end of Day 0, for the purposes of illustration in the figures.

## 3 Results

### 3.1 Controls

**Figure 1:**
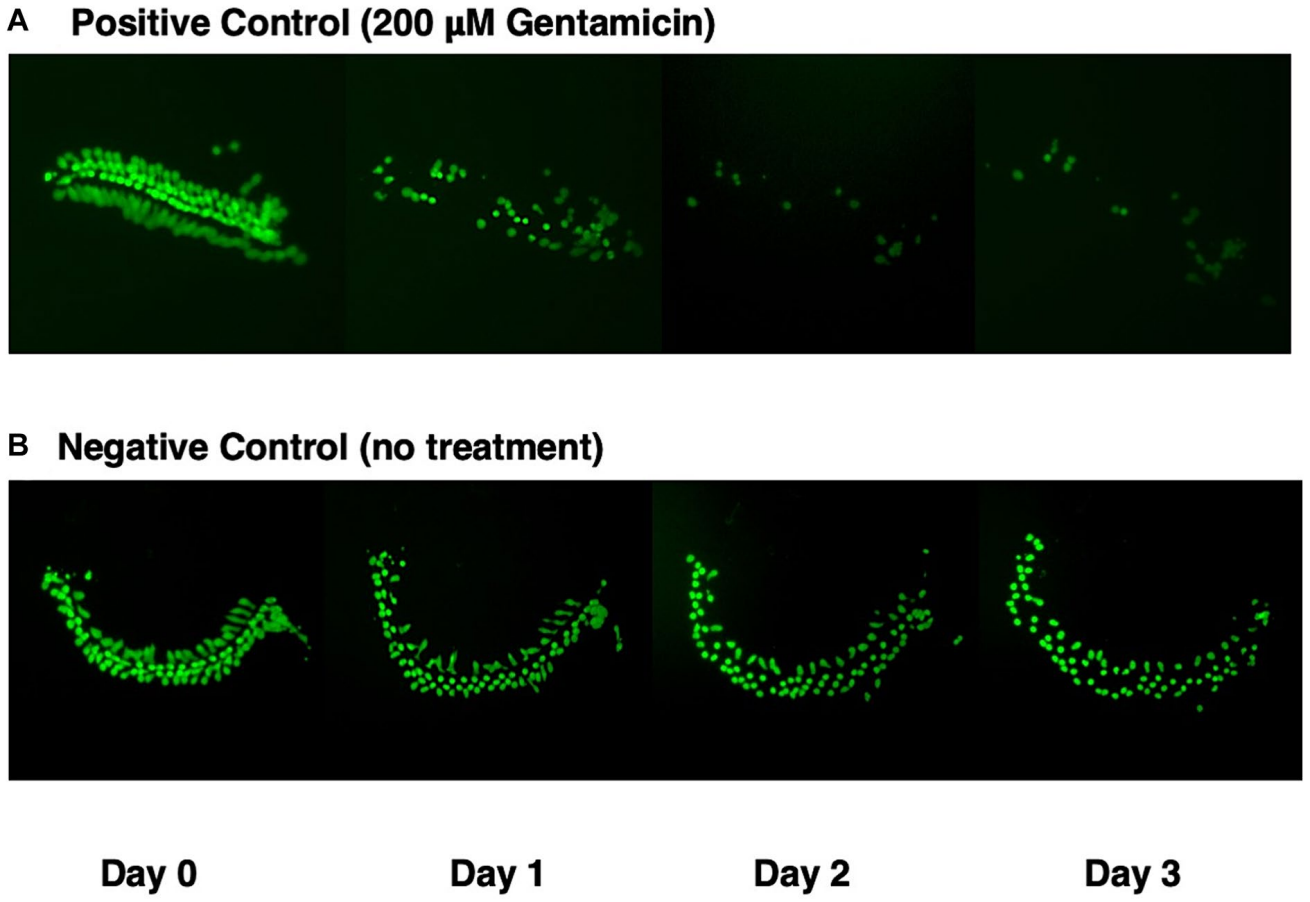
GFP expression (green) in micro-explants made from the isolated cochlea of transgenic *pou4f3*/*eGFP* mice. **(A)** Positive control explant treated with 200 μM gentamicin at end of Day 0, and maintained in culture for four days. **(B)** Negative control micro-explant also maintained in culture for four days.

Figure 1 illustrates a representative micro-explant at the end of four days in culture. Negative and positive control explants were placed into culture in media alone for 24 h to allow for attachment (Day 0). Figure 1A shows a positive control explant treated with 200 μM gentamicin at the beginning of Day 1. Significant loss of outer HCs and a minor loss of inner HCs was observed at the end of Day 1. By Day 2, essentially all outer HCs and some inner HCs were missing. By Day 3, only a remnant of inner HCs remained. In Figure 1B, the negative control explant showed minimal loss of HCs on Days 1 and 2, but scattered primarily outer HCs were missing on Day 3.

**Figure 2:**
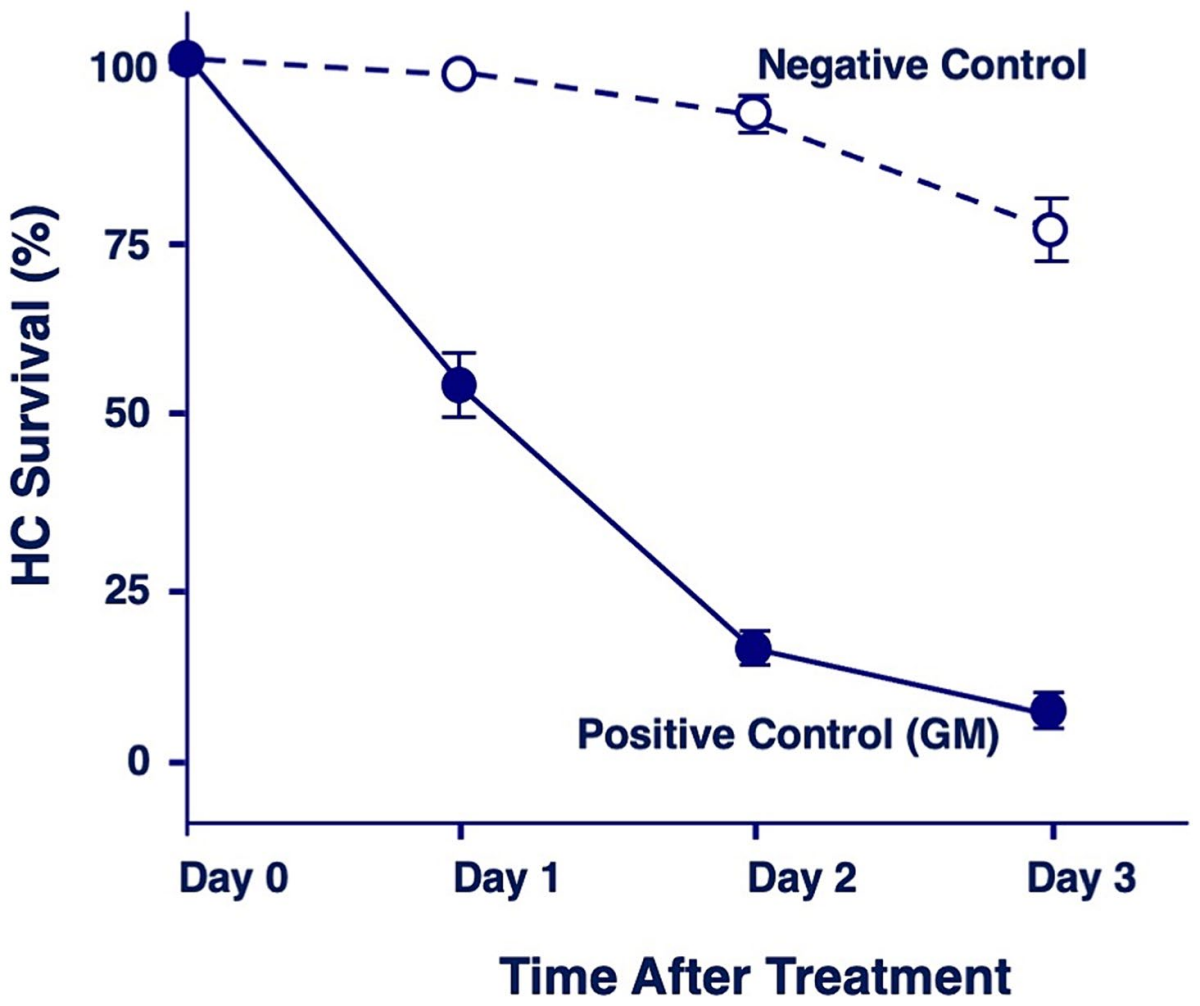
Quantitation of percentage of HCs remaining in negative control explants maintained in culture media alone, or in positive control explants after 1, 2 or 3 days of gentamicin treatment (200 μM).

Figure 2 illustrates the combined quantitative data from all negative and positive control micro-explants. Day 0 represents first day in culture but where the gentamicin treatment has not started yet. Negative controls showed the loss of a few percent of HCs on Day 1, 5–10% on Day 2 and 30% on Day 3. In contrast, positive explants lost 45% of HCs on Day 1, 94% on Day 2 and 96% on Day 3.

### 3.2 Compound combinations

**Figure 3:**
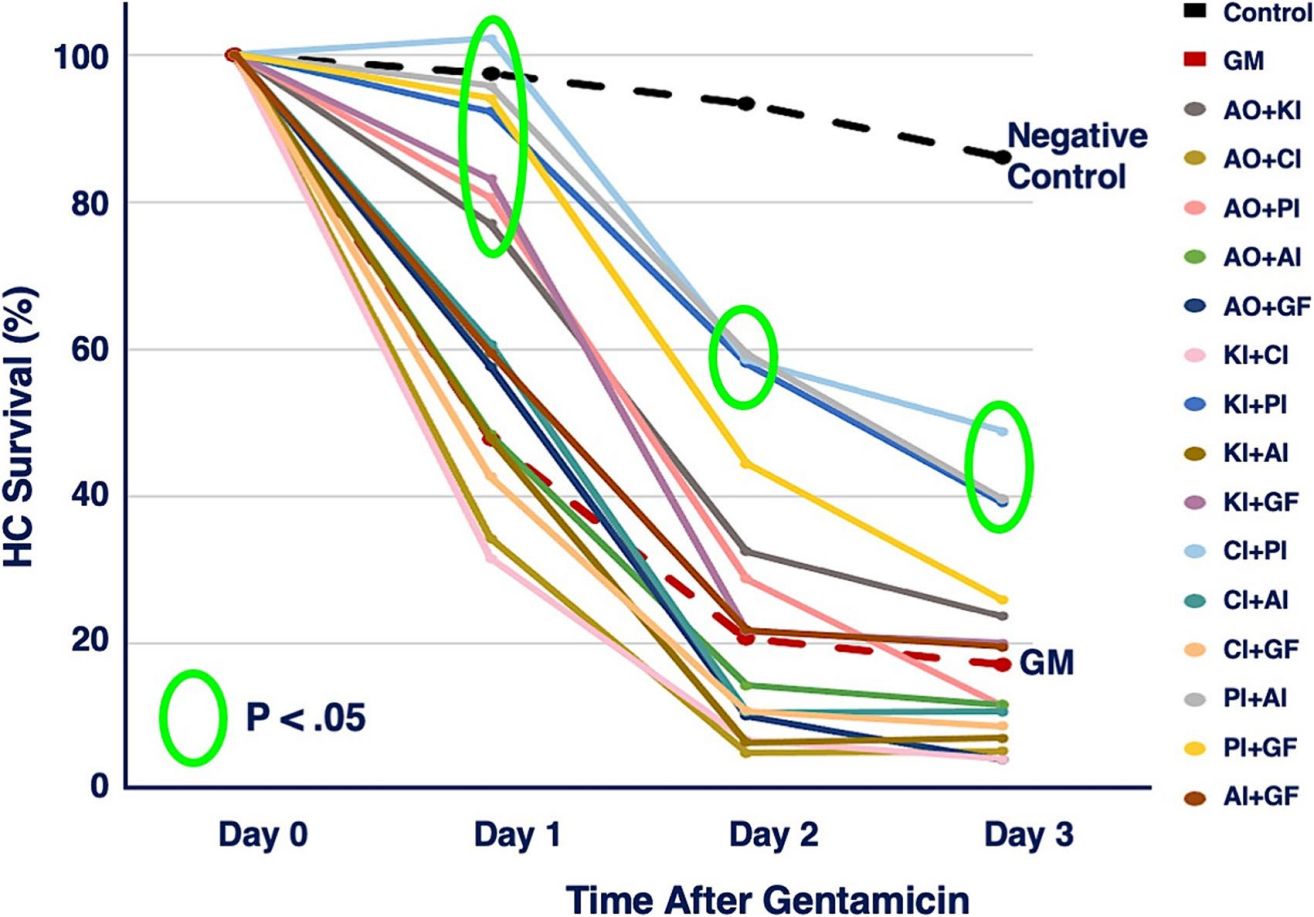
HC survival curves for all combinations of two factors pairs, before and for three days after co-treatment with gentamicin. Factors were provided at concentrations just below that which produces significant HC protection (Table 1). AO, antioxidant (NAC); KI, kinase inhibitor (CDTB); CI, calcium blocker (Nimodipine); PI, proliferation inhibitor (Fascaplysin); AI, apoptosis inhibitor (ZVAD-FMK); GF, growth factor (IGF-1).

**Figure 4:**
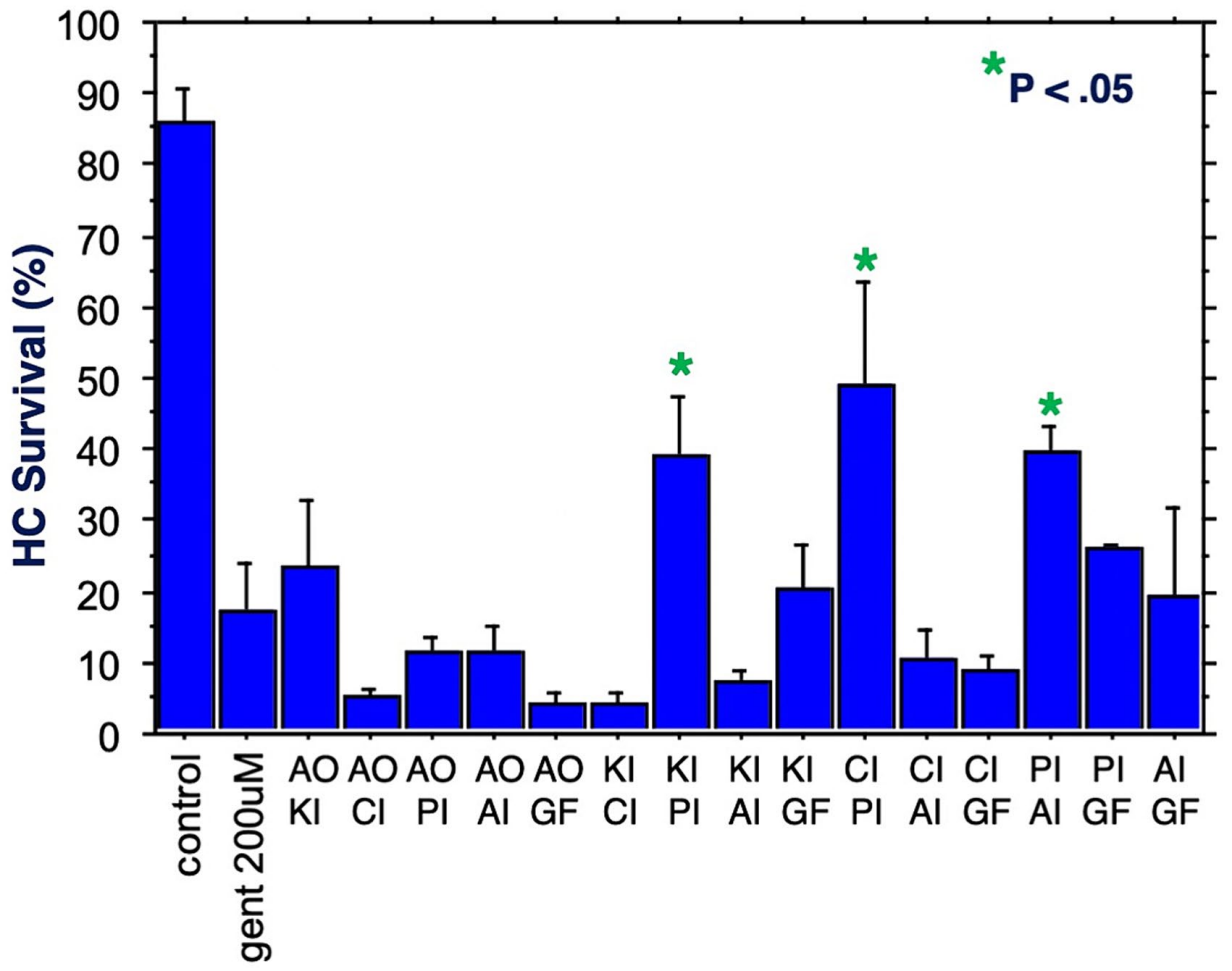
HC survival at the end of three days of gentamicin treatment for all combinations of two factors (*p < 0.05). AO, antioxidant; KI, kinase inhibitor; CI, calcium blocker; PI, proliferation inhibitor; AI, apoptosis inhibitor; GF, growth factor.

The six compounds used resulted in 15 unique combinations of two factors. Figure 3 shows the survival curves for all two-compound combinations. Seven of these pair combinations showed significant protection compared to gentamicin-treated micro-explants at the end of Day 1 post gentamicin treatment. This decreased to three at the end of Days 2 and 3. Figure 4 further illustrates HC survival on Day 3 for all combinations of 2-pair factors. Significant protection was noted for three of the pair combinations. The proliferation inhibitor was present in all three, in binary combination with the kinase inhibitor, the apoptosis inhibitor or the calcium channel blocker, with the latter resulting in 49% HC survival on Day 3. Compared to gentamicin alone, this represented an increase in survival by 2.7 fold.

**Figure 5:**
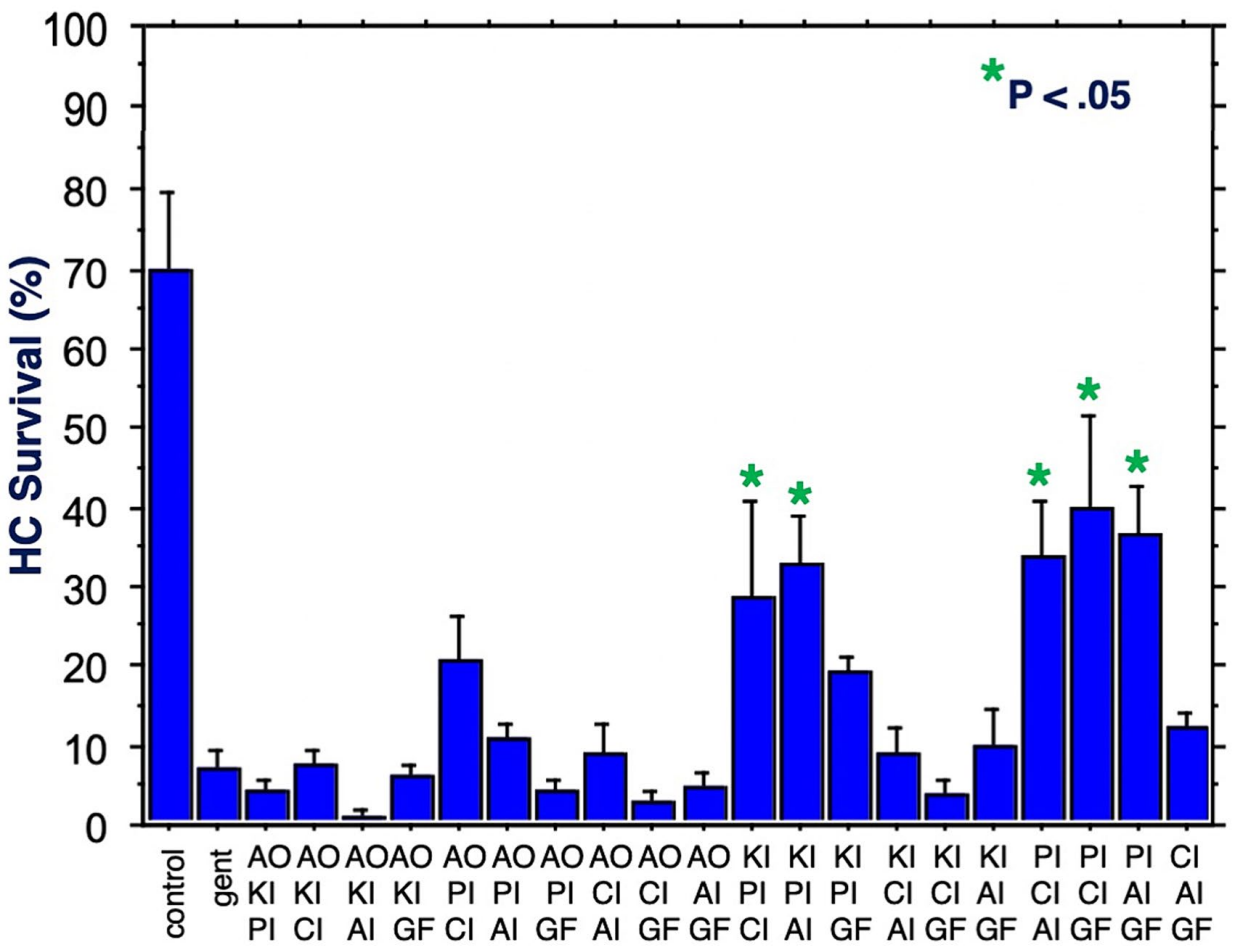
HC survival at the end of three days of gentamicin treatment for all combinations of three factors (*p < 0.05). AO, antioxidant; KI, kinase inhibitor; CI, calcium blocker; PI, proliferation inhibitor; AI, apoptosis inhibitor; GF, growth factor.

Figure 5 shows HC survival on Day 3 for all 20 combinations of three factors. Five combinations produced significant protection. All five included the proliferation inhibitor. Three protective combinations included the calcium channel blocker, three the apoptosis inhibitor, two the kinase inhibitor, two the growth factor, but none the antioxidant. The maximum protection achieved was 40%. Compared to gentamicin alone, this represented an increase in survival by 5.5 fold.

**Figure 6:**
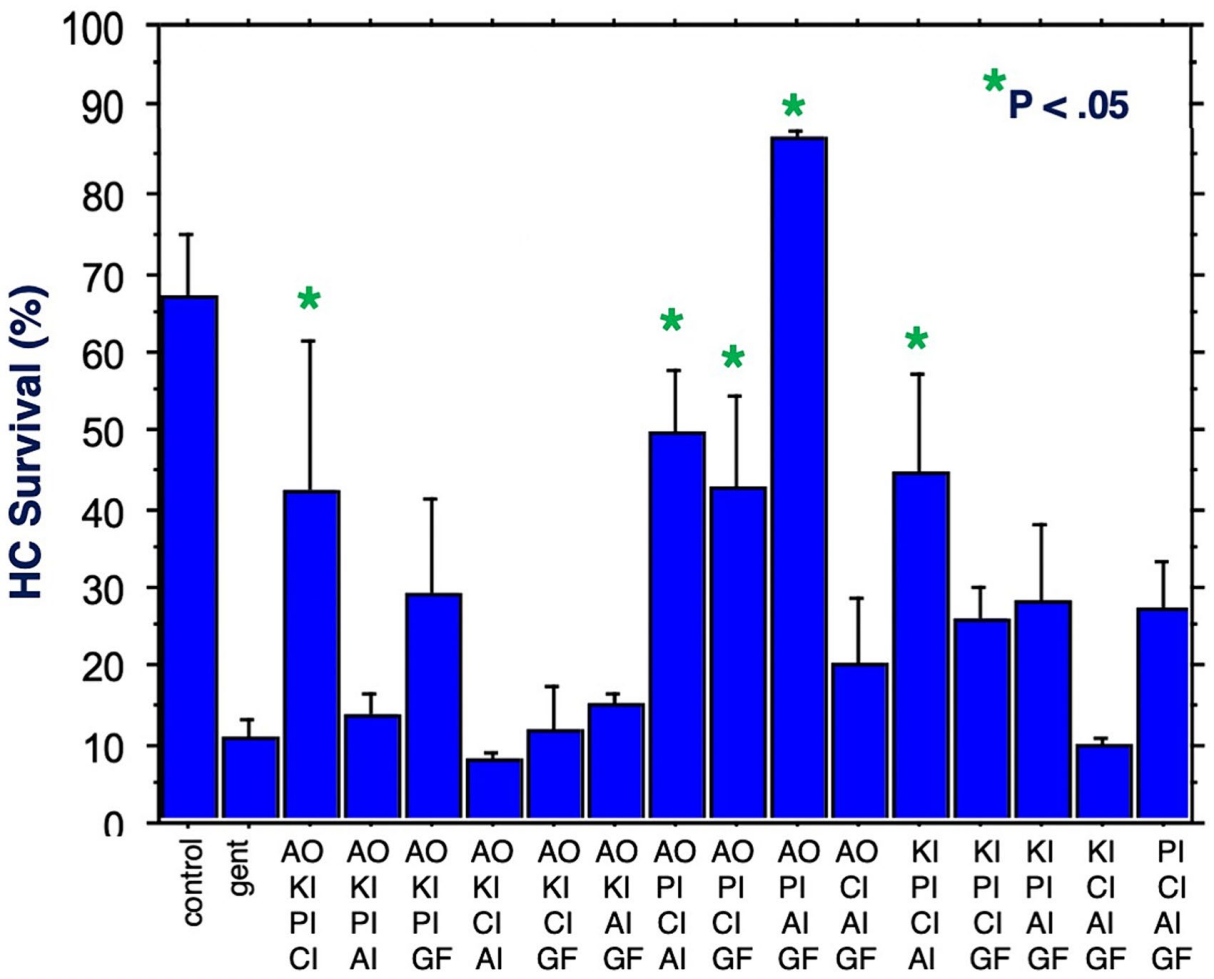
HC survival at the end of three days of gentamicin treatment for all combinations of four factors (*p < 0.05). AO, antioxidant; KI, kinase inhibitor; CI, calcium blocker; PI, proliferation inhibitor; AI, apoptosis inhibitor; GF, growth factor.

Figure 6 illustrates HC survival on Day 3 for all 15 combinations of four factors. Five combinations achieved significant protection against gentamicin. Again, all five included the proliferation inhibitor. Four included the calcium channel blocker, four the antioxidant, three the apoptosis inhibitor, two the growth factor and two the kinase inhibitor. The maximum protection achieved was 87%, by the combination of the proliferation inhibitor, the antioxidant, the apoptosis inhibitor and the growth factor. Compared to gentamicin alone, this represented an increase in survival by 7.9 fold.

**Figure 7:**
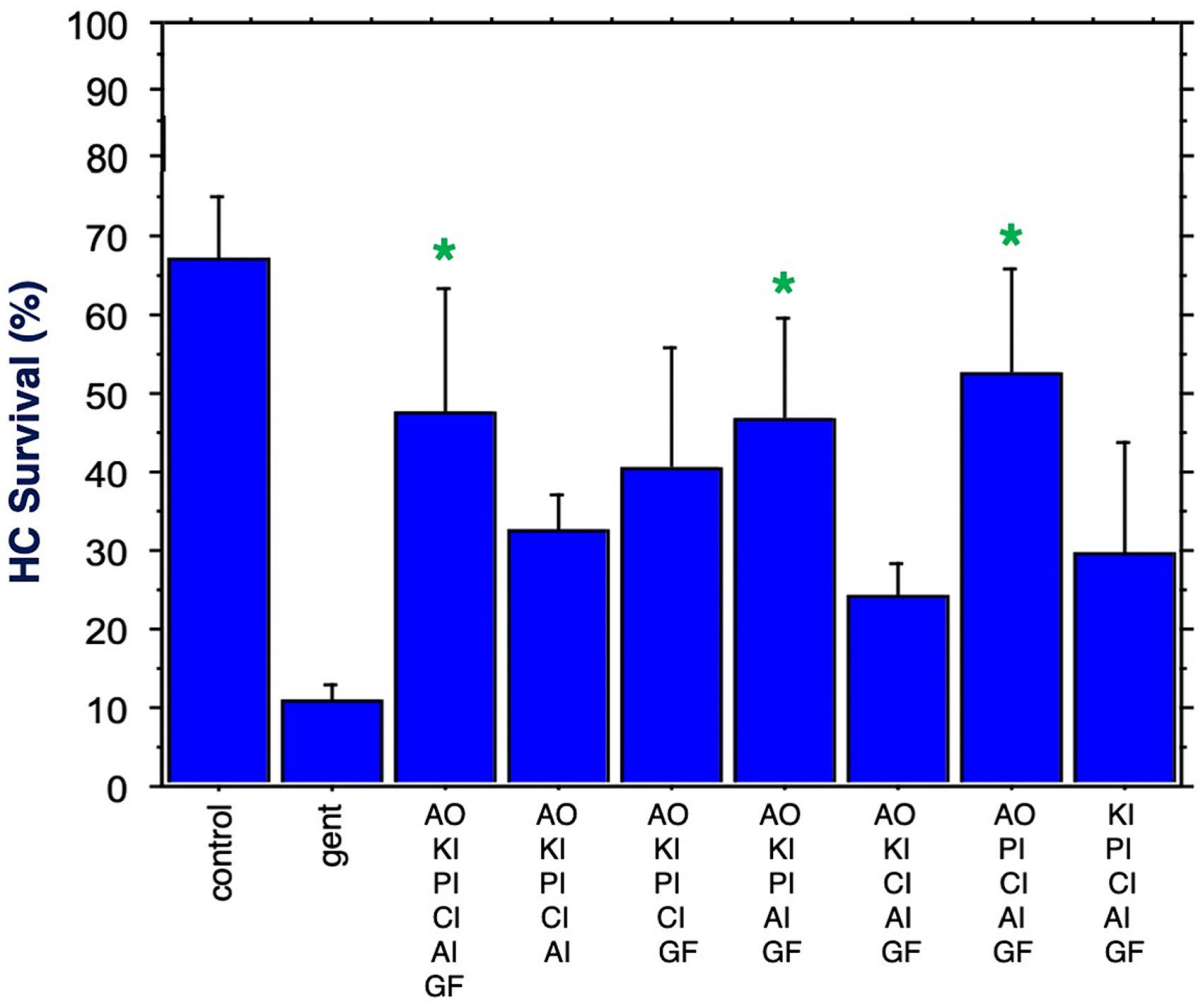
HC survival at the end of three days of gentamicin treatment for all combinations of five factors as well as all six factors (*p < 0.05). AO, antioxidant; KI, kinase inhibitor; CI, calcium blocker; PI, proliferation inhibitor; AI, apoptosis inhibitor; GF, growth factor.

Figure 7 shows HC survival achieved by the six combinations of five factors, plus by all six factors combined. Two of the five-compound combinations were significantly protective. One of these lacked the calcium channel blocker, while the other lacked the kinase inhibitor. The maximum HC protection of 52% was observed for the combination of the antioxidant, the proliferation inhibitor, the calcium channel blocker, the apoptosis inhibitor and the growth factor. Compared to gentamicin alone, this represented an increase in survival by 5.2 fold. All six compounds resulted in 47% HC survival at Day 3. Compared to gentamicin alone, this represented an increase in survival by 4.7 fold.

## 4 Discussion

### 4.1 Protective effects of factor combinations on HC survival

The results indicate that combinations of factors targeting different processes related to HC damage, delivered individually at sub-protective concentrations, interact to produce protection. The most protective combination of two factors increased the number of HCs surviving after three days of gentamicin by 2.8 times. The best combination of three factors increased HC survival by 5.5 times, and for four factors by 7.9 times. Survival enhancement then decreased to 4.7 times for the best five-factor combination, and by 4.4 times for all six factors. Increasing the number of factors also enhanced the percentage of combinations that were protective.

Experimental combinatorial studies have been recognized as useful to identify optimal combinations for the treatment of many disorders (Editorial, 2017), including cancer, (Lopez and Banerji, 2017; Wei et al., 2024) prior to any clinical trials. However, translating of the identified combination of treatments into clinical use would face significant difficulties. Translation from *in vitro* to *in vivo*, and from animal to human, are well-known hurdles (Van der Worp et al., 2010). Even if these barriers can be crossed, the use of the best factor combination in patients would require cochlear safety trials for each factor separately, and possibly trials for different factor combinations, in addition to the final four-factor combination (Patera et al., 2023). These difficulties make the use of the treatment combinations identified here for preventing HC loss in patients a distant goal. The value of the present study is rather to identify cellular processes that contribute to HC damage and protection, to provide evidence regarding their relative importance, and to document interactions.

It is immediately clear from the results that prevention of HCs from entering the cell cycle was a critical factor in preventing HC loss. The role of cell cycle inhibition in HC survival well known from studies of HC regeneration (Groves, 2010), and is mediated by the expression of the cyclin-dependent kinase inhibitor INK4dD (Chen et al., 2003). However, its importance in ototoxicity has not been as well recognized. All three of the combinations of two compounds that remained significantly protective through Day 3 included the cell proliferation inhibitor fascaplysin, as did the five protective combinations of three factors, the five protective combinations of four compounds, the two protective combinations of five factors, and of course the combination of all compounds. These data support a major role for induction of the cell cycle in ototoxic HC damage.

Three out of five protective combinations of three factors included the calcium channel inhibitor nimodipine, as did four out of the five protective four-factor combinations, and one of the five-factor combinations. Calcium increases in mitochondria are well-known to be a critical component of aminoglycoside-induced HC loss (Esterberg et al., 2014).

For the four-factor combinations, the most protective combination identified consisted of the proliferation inhibitor fascaplysin, the apoptosis inhibitor ZVAD-FMK, the growth factor IGF-1, and the antioxidant NAC. Fascaplysin, ZVAD-FMK, and IGF-1 formed a three-factor combination that was highly protective, and the inclusion of NAC appeared to enhance this protective effect. Adding additional factors to form a five or six factor combination, such as the calcium channel inhibitor nimodipine or the kinase inhibitor CDTB, maintained a strong protective effect but did not appear to enhance it further. The specific reasons for this outcome remain unclear, since nimodipine was often present in the protective combinations. The addition of these extra factors might disrupt critical cellular processes, as exemplified by CDTB, which inactivate Rho and related Rho family small GTPases (Bodmer et al., 2002).

### 4.2 Complexity of interactions in HC damage and protection

The interactions of cell damage and survival processes are highly complex, thus there are many potential means of interaction between the inhibitors and the growth factor used in this study. For example, cytoplasmic calcium is an important regulator of numerous cellular processes, thus understanding its role in any given situation is complex. However, increased levels of Ca^++^ are well known to stimulate cell division and/or apoptosis (e.g. Zhang et al., 2019), both of which are important to hair cell survival (e.g. Esterberg et al., 2014; Wu et al., 2020). By limiting Ca^++^ influx from the extracellular medium via L-type channels, nimodipine has the potential to potentiate the effects of the apoptosis inhibitor ZVA-FMK and the cell division inhibitor fascaplysin. At low levels, ROS and calcium interact to regulate cell homeostasis. However, higher levels of ROS can target ER-based calcium channels, leading to increased release of calcium and additional increases in ROS levels. Increased ROS and calcium can open the mitochondrial permeability transition pore, resulting in the release of pro-apoptotic factors (Görlach et al., 2015). Antioxidants can thus potentially interact with nimodipine and/or ZVAD-FMK. Growth factors stimulate cell survival signaling, including via ERK and AKT signaling to activate survival factors such as IAP (inhibitor of apoptosis) proteins (Vasudevan and Ryoo, 2015), and Bcl family members (Qian et al., 2022), providing a mechanism of for potentiation of ZVAD-FMK by IGF-1. The protein kinase inhibitor CDTB blocks the activation of Cd42, which is in the pathway linking K-Ras to activation of the transcriptional regulator JNK. The JNK pathway can also be activated by ROS. JNK can mediate in the increased expression of pro-apoptotic genes including as TNF-α, Fas-L and Bak (Dhanasekaran and Reddy, 2017). Thus, NAC or CDTB could potentially interact with ZVAD-FMK. Given this level of complexity, we can only speculate on the contributions of these and other possible interactions to HC protection from gentamicin.

### 4.3 Study limitations

There are a number of limitations of this study that must be considered in interpreting the results. First, the comparisons as mentioned above are only observational. The comparison across numbers of compounds were not statistically analyzed given the variability in controls. We acknowledge that differences in HC survival rates across these combinations could introduce biological variability that may affect the interpretation of our results.

We used a single low dose of each factor to allow the detection of interactive protection. Higher doses may well have produced different interactions. In particular, stronger drug dosages might increase the level of protection for some combinations, or decrease the potential for protection due to enhanced disturbance of critical cellular processes. Using multiple doses would have greatly expanded the scope of this project, especially if different dosages had been compared across compounds. Future studies will be required to use a range of dosages, to investigate these potential changes in interactions.

The decline in HC protection observed for combinations over time in culture (e.g. Figure 3) could reflect either increased damage from gentamicin and/or a decline in the concentrations of compounds. Given the volume difference between the culture well and the explant, and the relatively stable nature of the small molecule compounds employed (Elmlinger et al., 2005; Friciu et al., 2021; Green et al., 2004; Bharate et al., 2012), we would not expect significant changes in their levels over time in culture. Also, in the absence of proteases in the culture media, we would also not expect meaningful peptide degradation of IGF-1, CDTB or ZVAD-FMK. However, although we assume that compound concentrations did not change significantly over time in culture, it still remains a possibility and thus compound concentration changes remain a consideration when interpreting our data.

While damage to HCs from gentamicin decreases HC GFP^+^ fluorescence (see Figure 1), it is also possible that compounds or combinations could decrease GFP expression sufficiently to mimic HC loss. We think that if this occurred, it would most likely reflect additional HC damage rather than an effect limited to GFP. However, we did not see noticeable decreases in GFP fluorescence beyond those induced by gentamicin alone in our compound groups.

The *in vitro* method employed may well differ from that occurring *in vivo*. We studied immature hair cells, while most forms of ototoxic drug damage occurs in adults. The use of alternative modulators of each cellular process could also have affected the outcome. It should also be noted that this basic science study was not intended to lead to a human treatment. Rather, it was performed to identify critical processes in the hair cell damage mechanism and their relationships.

Finally, almost all pharmacological inhibitors affect additional cell processes, often in a dose-dependent manner. While the low dosages used were intended to minimize this issue, inhibition of an alternative cell process than the one intended could have affected HC protection.

## 5 Conclusion

The results support our prediction that combining protective factors would yield enhanced protection of HCs from ototoxicity. They also implicated cell division, ROS, apoptosis and protective growth factors as critical regulators of HC death, while calcium regulation and stress kinases appeared to be less important. The reduced relative protection observed with added factors above the optimal four-compound combination may have been the result of interfering with too many cellular processes, which itself could impair HC survival.

## Data Availability

The original contributions presented in the study are included in the article/Supplementary material, further inquiries can be directed to the corresponding author.
