# Supplementary material for: Combinatorial protection of cochlear hair cells: not too little but not too much
Source: Front Cell Neurosci. 2024 Sep 17;18:1458720. doi: 10.3389/fncel.2024.1458720 (PMC11442228; doi:10.3389/fncel.2024.1458720)
Supplement: Supplementary file 1 [file Table_1.DOCX]

**Supplementary Table 1. Two-compound Combinations**

| **Day 2** | **HCs mean** | **SEM** | **P value vs Gent** |
| --- | --- | --- | --- |
| Control | 97.5 | 0.7 | 0.000 S |
| Gent 200 µM | 47.7 | 7.4 | ----------- |
| AO/KI | 77.0 | 4.1 | 0.04 S |
| AO/CI | 34.2 | 2.6 | 0.335 |
| AO/PI | 80.6 | 5.6 | 0.015 S |
| AO/AI | 48.4 | 2.6 | 0.960 |
| AO/GF | 57.7 | 13.1 | 0.474 |
| KI/CI | 31.5 | 6.1 | 0.250 |
| KI/PI | 93.3 | 2.3 | 0.003 S |
| KI/AI | 47.1 | 3.5 | 0.987 |
| KI/GF | 83.2 | 21.1 | 0.009 S |
| CI/PI | 102.2 | 8.4 | 0.000 S |
| CI/AI | 60.7 | 13.9 | 0.352 |
| CI/GF | 42.7 | 4.3 | 0.701 |
| PI/AI | 95.8 | 6.9 | 0.001 S |
| PI/GF | 94.1 | 0.7 | 0.002 S |
| AI/GF | 59.5 | 12.3 | 0.398 |

| **Day 3** | **HCs Mean** | **SEM** | **P value vs Gent** |
| --- | --- | --- | --- |
| Control | 93.3 | 6.1 | 0.0001 S |
| Gent 200 µM | 20.7 | 7.7 | ---------- |
| AO/KI | 32.5 | 9.8 | 0.467 |
| AO/CI | 5.07 | 1.9 | 0.190 |
| AO/PI | 28.7 | 10.1 | 0.490 |
| AO/AI | 14.2 | 5.4 | 0.535 |
| AO/GF | 10.1 | 2.0 | 0.153 |
| KI/CI | 6.9 | 1.8 | 0.158 |
| KI/PI | 58.1 | 7.2 | 0.018 S |
| KI/AI | 6.6 | 1.2 | 0.266 |
| KI/GF | 21.7 | 7.4 | 0.730 |
| CI/PI | 58.6 | 12.3 | 0.001 S |
| CI/AI | 10.6 | 3.9 | 0.468 |
| CI/GF | 10.8 | 2.2 | 0.318 |
| PI/AI | 59.3 | 4.2 | 0.016 S |
| PI/GF | 44.4 | 3.4 | 0.333 |
| 21.8 | 21.8 | 12.9 | 0.797 |

| **Day 4** | **HCs Mean** | **SEM** | **P value vs Gent** |
| --- | --- | --- | --- |
| Control | 86.0 | 4.5 | 0.0001 S |
| Gent 200 µM | 17.2 | 7.7 | ---------- |
| AO/KI | 23.7 | 9.2 | 0.467 |
| AO/CI | 5.3 | 0.8 | 0.190 |
| AO/PI | 11.4 | 2.2 | 0.490 |
| AO/AI | 11.6 | 3.5 | 0.535 |
| AO/GF | 4.2 | 1.3 | 0.153 |
| KI/CI | 4.3 | 1.2 | 0.158 |
| KI/PI | 39.1 | 8.1 | 0.018 S |
| KI/AI | 7.2 | 1.8 | 0.266 |
| KI/GF | 20.1 | 6.5 | 0.730 |
| CI/PI | 48.8 | 14.6 | 0.001 S |
| CI/AI | 10.6 | 3.7 | 0.468 |
| CI/GF | 8.8 | 2.2 | 0.318 |
| PI/AI | 39.6 | 3.5 | 0.016 S |
| PI/GF | 25.9 | 3.4 | 0.333 |
| KI/CI | 19.5 | 12.9 | 0.797 |
