# Supplementary material for: Combinatorial protection of cochlear hair cells: not too little but not too much
Source: Front Cell Neurosci. 2024 Sep 17;18:1458720. doi: 10.3389/fncel.2024.1458720 (PMC11442228; doi:10.3389/fncel.2024.1458720)
Supplement: Supplementary file 2 [file Table_2.docx]

**Supplementary Table 2. Three-compound Combinations**

| **Day 2** | **HCs mean** | **SEM** | **P value vs Gent** |
| --- | --- | --- | --- |
| Control | 100.0 | 6.9 | 0.000 S |
| Gent 200 µM | 52.6 | 17.5 | ----------- |
| AO/KI/PI | 88.3 | 3.4 | 0.004 S |
| AO/KI/CI | 46.5 | 10.9 | 0.598 |
| AO/KI/AI | 38.9 | 3.3 | 0.240 |
| AO/KI/GF | 59.9 | 7.9 | 0.530 |
| AO/PI/CI | 95.7 | 2.0 | 0.001 S |
| AO/PI/AI | 95.7 | 1.9 | 0.001 S |
| AO/PI/GF | 90.0 | 7.9 | 0.002 S |
| AO/CI/AI | 52.7 | 6.8 | 0.990 |
| AO/CI/GF | 39.3 | 11.0 | 0.255 |
| AO/AI/GF | 57.3 | 10.8 | 0.692 |
| KI/PI/CI | 75.6 | 11.1 | 0.053 S |
| KI/PI/AI | 91.5 | 3.7 | 0.002 S |
| KI/CI/AI | 86.7 | 6.6 | 0.005 S |
| KI/CI/GF | 63.3 | 14.1 | 0.362 |
| KI/AI/GF | 42.2 | 2.8 | 0.370 |
| PI/CI/AI | 91.7 | 6.5 | 0.382 |
| PI/CI/GF | 91.1 | 3.9 | 0.002 S |
| PI/AI/GF | 94.2 | 4.0 | 0.001 S |
| CI/AI/GF | 52.3 | 5.7 | 0.973 |

| **Day 3** | **HCs Mean** | **SEM** | **P value vs Gent** |
| --- | --- | --- | --- |
| Control | 85.4 | 8.1 | <0.0001 S |
| Gent 200 µM | 9.3 | 2.1 | ----------- |
| AO/KI/PI | 32.8 | 1.9 | 0.015 S |
| AO/KI/CI | 8.7 | 2.5 | 0.954 |
| AO/KI/AI | 4.5 | 1.5 | 0.611 |
| AO/KI/GF | 9.4 | 2.7 | 0.986 |
| AO/PI/CI | 43.9 | 12.3 | 0.001 S |
| AO/PI/AI | 37.3 | 6.4 | 0.004 S |
| AO/PI/GF | 19.8 | 9.2 | 0.263 |
| AO/CI/AI | 12.8 | 2.7 | 0.706 |
| AO/CI/GF | 4.9 | 1.9 | 0.645 |
| AO/AI/GF | 10.6 | 3.1 | 0.889 |
| KI/PI/CI | 47.9 | 13.6 | 0.000 S |
| KI/PI/AI | 61.8 | 8.1 | 0.000 S |
| KI/PI/GF | 42.4 | 7.1 | 0.001 S |
| KI/CI/AI | 12.1 | 3.8 | 0.762 |
| KI/CI/GF | 6.2 | 1.4 | 0.741 |
| KI/AI/GF | 11.3 | 3.9 | 0.825 |
| PI/CI/AI | 43.8 | 8.2 | 0.001 S |
| PI/CI/GF | 62.0 | 12.5 | 0.000 S |
| PI/AI/GF | 69.0 | 2.2 | 0.000 S |
| CI/AI/GF | 17.3 | 1.8 | 0.389 |

| **Day 4** | **HCs Mean** | **SEM** | **P value vs Gent** |
| --- | --- | --- | --- |
| Control | 69.8 | 9.8 | 0.000 S |
| Gent 200 µM | 6.9 | 2.4 | ----------- |
| AO/KI/PI | 4.3 | 1.3 | 0.728 |
| AO/KI/CI | 5.6 | 2.0 | 0.937 |
| AO/KI/AI | 0.9 | 0.9 | 0.423 |
| AO/KI/GF | 5.9 | 1.4 | 0.891 |
| AO/PI/CI | 20.8 | 5.7 | 0.072 |
| AO/PI/AI | 10.6 | 2.2 | 0.630 |
| AO/PI/GF | 4.2 | 1.3 | 0.715 |
| AO/CI/AI | 9.1 | 3.5 | 0.781 |
| AO/CI/GF | 2.7 | 1.6 | 0.572 |
| AO/AI/GF | 28.7 | 11.8 | 0.762 |
| KI/PI/CI | 32.7 | 6.2 | 0.006 S |
| KI/PI/AI | 19.0 | 2.2 | 0.001 S |
| KI/CI/AI | 8.7 | 3.4 | 0.115 |
| KI/CI/GF | 3.7 | 1.7 | 0.820 |
| KI/AI/GF | 10.0 | 4.5 | 0.670 |
| PI/CI/AI | 33.7 | 7.0 | 0.001 S |
| PI/CI/GF | 39.7 | 11.9 | 0.000 S |
| PI/AI/GF | 36.6 | 6.3 | 0.000 S |
| CI/AI/GF | 12.3 | 1.6 | 0.484 |
