# Supplementary material for: Combinatorial protection of cochlear hair cells: not too little but not too much
Source: Front Cell Neurosci. 2024 Sep 17;18:1458720. doi: 10.3389/fncel.2024.1458720 (PMC11442228; doi:10.3389/fncel.2024.1458720)
Supplement: Supplementary file 3 [file Table_3.docx]

**Supplementary Table 3. Four-compound Combinations**

| **Day 2** | **HCs mean** | **SEM** | **P value vs Gent** |
| --- | --- | --- | --- |
| Control | 97.0 | 1.8 | 0.000 S |
| Gent 200 µM | 66.9 | 13.1 | ----------- |
| AO/KI/PI/CI | 94.2 | 1.5 | 0.008 S |
| AO/KI/PI/AI | 86.3 | 8.7 | 0.053 |
| AO/KI/PI/GF | 86.8 | 3.7 | 0.048 S |
| AO/KI/CI/AI | 64.8 | 16.2 | 0.829 |
| AO/KI/CI/GF | 79.6 | 2.0 | 0.199 |
| AO/KI/AI/GF | 50.3 | 1.6 | 0.095 |
| AO/PI/CI/AI | 86.6 | 2.3 | 0.050 S |
| AO/PI/CI/GF | 87.0 | 3.6 | 0.046 S |
| AO/PI/AI/GF | 96.8 | 6.8 | 0.004 S |
| AO/CI/AI/GF | 69.3 | 12.8 | 0.804 |
| KI/PI/CI/AI | 92.4 | 3.0 | 0.013 |
| KI/PI/CI/GF | 91.7 | 4.7 | 0.015 |
| KI/PI/AI/GF | 100 | 0.0 | 0.002 S |
| KI/CI/AI/GF | 47.8 | 1.7 | 0.057 |
| PI/CI/AI/GF | 96.2 | 1.4 | 0.005 S |

| **Day 3** | **HCs Mean** | **SEM** | **P value vs Gent** |
| --- | --- | --- | --- |
| Control | 83.2 | 1.8 | 0.0001 S |
| Gent 200 µM | 15.3 | 3.5 | ---------- |
| AO/KI/PI/CI | 58.0 | 3.4 | 0.001 S |
| AO/KI/PI/AI | 30.9 | 2.5 | 0.202 |
| AO/KI/PI/GF | 50.7 | 7.2 | 0.006 S |
| AO/KI/CI/AI | 16.6 | 6.5 | 0.921 |
| AO/KI/CI/GF | 21.7 | 8.8 | 0.599 |
| AO/KI/AI/GF | 17.1 | 1.8 | 0.883 |
| AO/PI/CI/AI | 74.2 | 0.7 | 0.000 S |
| AO/PI/CI/GF | 57.1 | 9.9 | 0.002 S |
| AO/PI/AI/GF | 91.4 | 1.5 | 0.000 S |
| AO/CI/AI/GF | 23.2 | 9.0 | 0.518 |
| KI/PI/CI/AI | 57.9 | 10.5 | 0.001 S |
| KI/PI/CI/GF | 39.6 | 10.5 | 0.051 |
| KI/PI/AI/GF | 57.4 | 12.1 | 0.001 S |
| KI/CI/AI/GF | 13.8 | 3.2 | 0.899 |
| PI/CI/AI/GF | 36.2 | 8.5 | 0.090 |

| **Day 4** | **HCs Mean** | **SEM** | **P value vs Gent** |
| --- | --- | --- | --- |
| Control | 67.2 | 7.7 | 0.0001 S |
| Gent 200 µM | 11.0 | 2.1 | ---------- |
| AO/KI/PI/CI | 42.1 | 19.2 | 0.014 S |
| AO/KI/PI/AI | 13.6 | 2.8 | 0.827 |
| AO/KI/PI/GF | 29.0 | 12.0 | 0.142 |
| AO/KI/CI/AI | 8.0 | 1.0 | 0.803 |
| AO/KI/CI/GF | 11.7 | 5.8 | 0.954 |
| AO/KI/AI/GF | 15.0 | 1.3 | 0.738 |
| AO/PI/CI/AI | 49.6 | 8.0 | 0.003 S |
| AO/PI/CI/GF | 42.7 | 11.9 | 0.012 S |
| AO/PI/AI/GF | 87.2 | 0.8 | 0.000 S |
| AO/CI/AI/GF | 20.2 | 8.6 | 0.448 |
| KI/PI/CI/AI | 44.3 | 12.9 | 0.009 S |
| KI/PI/CI/GF | 25.6 | 4.3 | 0.231 |
| KI/PI/AI/GF | 28.2 | 9.7 | 0.161 |
| KI/CI/AI/GF | 9.6 | 1.0 | 0.911 |
| PI/CI/AI/GF | 27.1 | 6.1 | 0.187 |
