# Supplementary material for: Combinatorial protection of cochlear hair cells: not too little but not too much
Source: Front Cell Neurosci. 2024 Sep 17;18:1458720. doi: 10.3389/fncel.2024.1458720 (PMC11442228; doi:10.3389/fncel.2024.1458720)
Supplement: Supplementary file 4 [file Table_4.docx]

**Supplementary Table 4. Five- and Six-compound Combinations**

**Five-compound Combinations**

| **Day 2** | **HCs mean** | **SEM** | **P value vs Gent** |
| --- | --- | --- | --- |
| Control | 97.0 | 1.8 | 0.000 S |
| Gent 200 µM | 66.9 | 13.1 | ----------- |
| AO/KI/PI/CI/AI | 96.5 | 0.7 | 0.001 S |
| AO/KI/PI/CI/GF | 96.1 | 2.6 | 0.001 S |
| AO/KI/PI/AI/GF | 96.6 | 1.8 | 0.001 S |
| AO/KI/CI/AI/GF | 75.6 | 5.9 | 0.234 |
| AO/PI/CI/AI/GF | 98.6 | 0.8 | 0.000 S |
| KI/PI/CI/AI/GF | 98.8 | 1.2 | 0.000 S |

| **Day 3** | **HCs Mean** | **SEM** | **P value vs Gent** |
| --- | --- | --- | --- |
| Control | 97.0 | 1.8 | <0.0001 S |
| Gent 200 µM | 15.3 | 3.5 | ----------- |
| AO/KI/PI/CI/AI | 53.4 | 8.0 | 0.011 S |
| AO/KI/PI/CI/GF | 55.4 | 13.9 | 0.008 S |
| AO/KI/PI/AI/GF | 63.6 | 9.4 | 0.002 S |
| AO/KI/CI/AI/GF | 25.8 | 7.0 | 0.448 |
| AO/PI/CI/AI/GF | 66.8 | 8.9 | 0.001 S |
| KI/PI/CI/AI/GF | 43.3 | 8.8 | 0.053 |

| **Day 4** | **HCs Mean** | **SEM** | **P value vs Gent** |
| --- | --- | --- | --- |
| Control | 67.2 | 7.7 | <0.0001 S |
| Gent 200 µM | 11.0 | 2.1 | ----------- |
| AO/KI/PI/CI/AI | 32.6 | 4.4 | 0.190 |
| AO/KI/PI/CI/GF | 40.6 | 15.2 | 0.079 |
| AO/KI/PI/AI/GF | 46.5 | 13.1 | 0.038 S |
| AO/KI/CI/AI/GF | 24.0 | 4.2 | 0.422 |
| AO/PI/CI/AI/GF | 52.4 | 13.6 | 0.018 S |
| KI/PI/CI/AI/GF | 29.5 | 14.1 | 0.258 |

**Six-compound Combination**

| **Day 2** | **HCs Mean** | **SEM** | **P value vs Gent** |
| --- | --- | --- | --- |
| AO/KI/PI/CI/AI/GF | 97.9 | 1.1 | 0.000 S |

| **Day 3** | **HCs Mean** | **SEM** | **P value vs Gent** |
| --- | --- | --- | --- |
| AO/KI/PI/CI/AI/GF | 56.3 | 16.1 | 0.007 S |

| **Day 4** | **HCs Mean** | **SEM** | **P value vs Gent** |
| --- | --- | --- | --- |
| AO/KI/PI/CI/AI/GF | 47.6 | 15.7 | 0.033 S |
